# A novel severity score to predict inpatient mortality in COVID-19 patients

**DOI:** 10.1038/s41598-020-73962-9

**Published:** 2020-10-07

**Authors:** David J. Altschul, Santiago R. Unda, Joshua Benton, Rafael de la Garza Ramos, Phillip Cezayirli, Mark Mehler, Emad N. Eskandar

**Affiliations:** 1 Department of Neurological Surgery, Montefiore Medical Center, 3316 Rochambeau Ave., Bronx, NY 10467 USA; 2 Department of Neurology, Montefiore Medical Center, Bronx, NY USA; 3 Leo M. Davidoff Department of Neurosurgery, Montefiore Medical Center, Bronx, NY USA; 4 Albert Einstein College of Medicine, Bronx, NY USA

**Keywords:** Outcomes research, Virology, Medical research, Prognostic markers

## Abstract

COVID-19 is commonly mild and self-limiting, but in a considerable portion of patients the disease is severe and fatal. Determining which patients are at high risk of severe illness or mortality is essential for appropriate clinical decision making. We propose a novel severity score specifically for COVID-19 to help predict disease severity and mortality. 4711 patients with confirmed SARS-CoV-2 infection were included. We derived a risk model using the first half of the cohort (n = 2355 patients) by logistic regression and bootstrapping methods. The discriminative power of the risk model was assessed by calculating the area under the receiver operating characteristic curves (AUC). The severity score was validated in a second half of 2356 patients. Mortality incidence was 26.4% in the derivation cohort and 22.4% in the validation cohort. A COVID-19 severity score ranging from 0 to 10, consisting of age, oxygen saturation, mean arterial pressure, blood urea nitrogen, C-Reactive protein, and the international normalized ratio was developed. A ROC curve analysis was performed in the derivation cohort achieved an AUC of 0.824 (95% CI 0.814–0.851) and an AUC of 0.798 (95% CI 0.789–0.818) in the validation cohort. Furthermore, based on the risk categorization the probability of mortality was 11.8%, 39% and 78% for patient with low (0–3), moderate (4–6) and high (7–10) COVID-19 severity score. This developed and validated novel COVID-19 severity score will aid physicians in predicting mortality during surge periods.

## Introduction

The first confirmed case of COVID-19 in New York City was on March 1st, 2020. Within a few short weeks all of the hospitals in the area were overwhelmed hitting a peak on April 6th, 2020 of 6,377 confirmed positive cases that day. As of July 3rd, 2020, there have been 18,535 deaths, 55,110 hospitalizations and a total of 213,212 cases in this city^1^. New York City is an international travel hub with a high population density, and a heavy reliance on mass transportation that provided the permissive substrate for rapid viral spread^2^. As such, this region was one of the earliest areas in the United States to encounter the full impact of the pandemic^3^. Over these first few months much has been learned about the disease, its deadliness, and those who are at higher risk for dying.

In many people the disease is mild and self-limiting, but in a considerable portion of patients the disease is severe and fatal. Determining which patients are at high risk of severe illness or mortality is an essential part of understanding this illness. Prior reports from Wuhan identified certain comorbidities as diabetes, hypertension and coronary artery disease as patients more likely to present to their hospital^4^. They also discovered that patients with older age, higher Sequential Organ Failure Assessment (SOFA) score, and elevated d-dimers were significantly associated with inpatient mortality^4^. Further reports have shown other predictors of poor outcome such as acute kidney injury, acute hepatic injury, the need for mechanical ventilation, elevated c-reactive protein (CRP), interleukin-6 (IL-6), lymphocyte count, and Procalcitonin levels^5–8^.

COVID-19 is unique in its ability to not only cause sepsis, and multi-system organ failure, but also to cause a severe inflammatory response that can lead to systemic multi-vascular thrombosis^9,10^. While the SOFA score is also predictive of mortality for COVID-19, it does not address the additional thrombotic mitigators of severe illness^11^. Other reports have recommended the use of the International Society of Thrombosis and Haemostasis (ISTH) Disseminated Intravascular Coagulation Score (DICS), which was initially developed to help predict the development of disseminated intravascular coagulation (DIC), and now being used to help guide the use of anti-coagulation for patients with COVID-19^12–14^. We propose a novel score specifically for COVID-19 in-hospital mortality, combining elements of both of these scores to help predict disease severity and mortality.

## Methods

After approval of this study by the Montefiore Medical Center/Albert Einstein College of Medicine Institutional Review Board, information on demographics, comorbidities, admission laboratory values, admission medications, admission supplemental oxygen orders, discharge and mortality was identified through a healthcare surveillance software package (Clinical Looking Glass [CLG]; Streamline Health, Atlanta, Georgia) and review of the primary medical records. The Montefiore Medical Center/Albert Einstein College of Medicine Institutional Review Board approved waiver of patient informed consent due to the retrospective design of the study. To our knowledge, a description of the entire cohort of patients, as in the current manuscript, has not been reported in other submissions. In the interest of transparency, anonymized data will be made available at https://figshare.com/s/79827c396af7df42b3d7.

All methods were carried out in accordance with relevant guidelines and regulations Laboratory measures were extracted by identifying those obtained-on-admission. Comorbidities were identified based on the International Coding Disease coding system (ICD-10). The comorbidities chosen for this study are those used in the Charlson Comorbidity Index. Each patient’s medical record was queried for any diagnosis occurring within 5 years of his or her index admission. We included the laboratory markers that were made part of the routine tests on admission during the period of the study in our institution, among the available markers we selected the ones that have been reported to be commonly altered accordingly to recent studies (Online Appendix 1).

This study is an observational cohort study validating a novel, simple COVID-19 in-hospital mortality score to predict inpatient mortality risk in 4711 patients with confirmed SARS-CoV-2 infection using a combination of presentation vital signs, and basic admission laboratory values. This model was created on patients presenting from March 1st to April 16th. We used the first numeric half of patients during this period (n = 2355) as the “derivation cohort” in which the severity score was developed and internally validated. The second numeric half of our cohort (n = 2356) was used to confirm the power of the prediction score; this part of the cohort was considered the “validation cohort”.

Inclusion criteria was defined as all patients admitted to a hospital within a large healthcare network that were positive by detection of SARS-CoV-2 RNA using real-time reverse transcriptase–polymerase chain reaction (RT-PCR) assay testing, performed within the hospital system or documented at an outside system prior to transfer. Patients evaluated in the emergency room but not admitted, or those that died in the emergency room, were excluded from the analysis, given the relative paucity of data. Most patients had only one admission, and we only considered the last hospitalization for those that had multiple admissions during this period.

### Statistical analysis

Continuous values were represented using mean ± standard deviation (SD), or median and interquartile range (IQR). Categorical variables were described using frequencies and proportions. Comparisons were performed using Student’s *t* test, the nonparametric Mann–Whitney test or χ2 tests as appropriate. No imputation was made for missing data. The primary outcome of this study was in-hospital mortality. Hence, all the following statistical steps were done with in-hospital mortality as the only dependent variable.

For easier application to a risk score model, when performing multiple regression analysis, most continuous variables were converted to categories based on published data as follows: advanced age (≥ 60 years, ≥ 70 years, and ≥ 80 years), body mass index (< 18.5 or > 24.9 kg/m^2^), oxygen saturation (< 94%), temperature (> 38 °C), mean arterial pressure (MAP < 80 mmHg, < 70 mmHg, < 60 mmHg), white blood cell count (< 4800 or > 10,800 per mm^3^), Lymphocytes (< 1000 per mm^3^), platelet count (≤ 150,000 per mm^3^), alanine aminotransferase (ALT > 40 U/L), aspartate aminotransferase (AST > 40 U/L), ferritin (> 300 µg/L), INR (> 1.2), d-dimer (> 3 mg/ml), creatinine (> 150 µmol/L), blood urea nitrogen (BUN) (> 35 mg/dL), glucose (< 60 or > 500 mg/dL), sodium (< 139 or > 154 mmol/L), interleukin-6 (IL-6) (> 150 pg/ml), C-reactive protein (CRP) (> 10 mg/L), Procalcitonin (> 0.1 ng/ml), and Troponin (> 0.1 ng/ml).

Candidate predictors with *P* < 0.10 in univariate analyses were included a multiple logistic regression. In addition, a backward stepwise bootstrap regression model, in which 1000 random samples patients were generated with replacement, was also performed to investigate the relative importance of each variable included in our model^15^. Frequencies of occurrence of each covariate in the final model were noted; if predictors occurred in 70% or more of the bootstrap models, they were retained in the final multiple regression model. Beta coefficients and odds ratios (OR) were calculated with 95% confidence intervals (CI). The multiple regression coefficients of the predictive factors were used to assign integer points for the prediction score. However, for the simplicity of the score we allocated points in sequential order for variables with multiple categories (e.g., age in years < 60, ≥ 60, ≥ 70, and ≥ 80 would equal to 0, 1, 2 and 3 points in the score, respectively).

As described in previous validation methods^13^, we assessed the discriminative power of the prediction score by calculating the area under the receiver operating characteristic (ROC) curves (AUC). A predictor with an AUC above 0.7 was considered to be useful, while an AUC between 0.8 and 0.9 indicated good diagnostic accuracy. Risk categories were determined using the classification and regression tree (CART) analysis. The CART algorithm builds decision tree based on Gini’s impurity index as splitting criterion; the score was iteratively subdivided to find the cut-off point that produces the greatest reduction of impurity, meaning that it measures how often a random patient that died will be incorrectly labeled as low-risk and vice versa, a patient that survived will be labeled as high-risk^16^. Calibration of the risk score reflecting the link between predicted and observed risk, was evaluated by the Hosmer–Lemeshow goodness of fit test. A *P* value < 0.05 was considered statistically significant for all analyses. Data were analyzed using the STATA version 12 and IBM SPSS version 24.

## Results

Distribution of socio-demographics, comorbidities, vital signs and laboratory values between the validation and derivation cohorts are shown in Table 1. A total of 2355 COVID-19 positive patients were treated in our hospital during the first half of period chosen during the New York City outbreak (derivation cohort), from which 621 (26.4%) patients died. The validation cohort consisted of 2356 COVID-19 positive patients out of which 527 (22.4%) died.

**Table 1:** Baseline characteristics of COVID-19 positive patients in the derivation and validations cohorts.

Baseline characteristics	Derivation cohort (n = 2355)	Validation cohort (n = 2356)
Age-years, mean (SD)	65.3 (15.9)	61.4 (17.2)
Female sex, n (%)	1256 (53.3)	944 (40.1)
White, n (%)	269 (11.4)	197 (8.4)
African American, n (%)	1011 (42.9)	732 (31.1)
Hispanic, n (%)	837 (35.5)	916 (38.9)
Asian, n (%)	44 (1.9)	77 (3.3)
Body mass index (kg/m^2^) (IQR)	28.9 (24.8–33.8)	28.1 (24.3–32.3)
Diabetes simple, n (%)	442 (18.8)	244 (10.4)
Diabetes complicated, n (%)	323 (13.7)	172 (7.3)
Congestive heart failure, n (%)	357 (15.2)	184 (7.8)
Myocardial infarction, n (%)	137 (5.8)	64 (2.7)
Chronic pulmonary disease, n (%)	181 (7.7)	84 (3.6)
Temperature (°C), (IQR)	37.1 (36.7–37.7)	37.1 (36.7–37.7)
Oxygen saturation (%), (IQR)	95 (90–98)	95 (90–98)
Mean Arterial Pressure (MAP)-mmHg, (IQR)	86.7 (75.7–95.3)	86.7 (76–96.7)
White Blood Cells (WBC) per mm^3^, (IQR)	7200 (5300–10,000)	7400 (5400–10,500)
Lymphocytes per mm^3^, (IQR)	1000 (700–1400)	1000 (700–1500)
Platelets k per mm^3^, (IQR)	212 (158–276)	211.5 (158–281.5)
Alanine aminotransferase (AST) U/L, (IQR)	25 (15–41)	27 (16–46)
Aspartate aminotransferase (ALT) U/L , (IQR)	37 (24–61)	38 (24–64)
Ferritin µg/L , (IQR)	778 (369–1623)	675 (236–1459)
International normalized ratio (INR), (IQR)	1.1 (1.0–1.2)	1.1 (1.0–1.2)
D-dimer mg/ml, (IQR)	1.24 (0.36–3.16)	1.12 (0.0–2.95)
Creatinine µmol/L, (IQR)	110 (80–190)	102 (76–170)
Blood urea nitrogen (BUN) mg/dL, (IQR)	18 (11–35)	16 (9–32)
Glucose mg/dL, (IQR)	117 (105–167)	116 (91–161)
Sodium mmol/L, (IQR)	137 (134–140)	137 (134–140)
Interleukin-6 (IL-6) pg/ml, (IQR)	40.7 (17.3–88.9)	36.1 (14.6–80.1)
C-Reactive protein (CRP) mg/L, (IQR)	6.1 (0.5–15.9)	7 (1.4–16.2)
Procalcitonin ng/ml, (IQR)	0.2 (0.1–1.0)	0.2 (0.09–0.7)
Troponin ng/ml, (IQR)	0.01 (0.01–0.02)	0.01 (0.01–0.03)
In-hospital mortality, n (%)	621 (26.4)	527 (22.4)

Missing data: Congestive heart failure (2%), Chronic Pulmonary Disease (3%), Oxygen saturation (4%), Temperature (3%), Mean arterial pressure (5%), D-dimer (21%), Platelets (3%), INR (9%), BUN (12%), Creatinine (3%), Sodium (4%), Glucose (29%), AST (5%), ALT (4%), WBC (3%), Lymphocytes (3%), IL-6 (66%), Ferritin (28%), CRP (14%), Procalcitonin (44%), and Troponin (14%).

The univariate analysis showed 22 potential predictors with a *P* < 0.1 (Table 2). Out of the 22 candidate predictors, 10 variables remained as independent predictors in the multiple logistic regression analysis, including age (> 60, > 70 and > 80 years), female sex, oxygen saturation < 94%, mean arterial pressure (MAP) (< 80, < 70 and < 60 mmHg), international normalized ratio (INR) > 1.2, creatinine > 150 µmol/L, blood urea nitrogen (BUN) > 30 mg/dL, interleukin-6 (IL-6) > 150 pg/ml mol/dL, C-reactive protein (CRP) > 10, and procalcitonin > 0.1 (Table 3).

**Table 2:** Univariate analysis of discharged and dead patients with Covid-19 in the derivation cohort.

Predictors	Discharged (n = 1733)	Died (n = 621)	*p* value
Age-years, mean (SD)	62.72 (16.1)	72.55 (13.1)	< 0.001
Female sex, n (%)	962 (55.5)	294 (47.3)	< 0.001
White, n (%)	188 (10.8)	81 (13)	0.139
African American, n (%)	759 (43.8)	252 (40.6)	
Hispanic, n (%)	626 (36.1)	211 (34)	
Asian, n (%)	27 (1.6)	17 (2.7)	
Body mass index (kg/m^2^) (IQR)	29.1 (25–33.9)	28.2 (23.6–33.2)	0.42
Diabetes simple, n (%)	327 (18.9)	115 (18.5)	0.852
Diabetes complicated, n (%)	241 (13.9)	82 (13.2)	0.666
Congestive heart failure, n (%)	248 (14.3)	109 (17.6)	0.053
Myocardial infarction, n (%)	102 (5.9)	35 (5.6)	0.822
Chronic pulmonary disease, n (%)	120 (6.9)	61 (9.8)	0.02
Temperature (°C), (IQR)	37.06 (36.7–37.67)	37.17 (36.72–37.8)	0.001
Oxygen saturation (%), (IQR)	95 (92–98)	92 (84–96)	< 0.001
Mean Arterial Pressure (MAP)-mmHg, (IQR)	89 (80–96.7)	72.3 (53.3–88.3)	< 0.001
White Blood Cells per mm^3^, (IQR)	7 (5.2–9.5)	8.1 (5.8–11.6)	0.041
Lymphocytes per mm^3^, (IQR)	1 (0.7–1.4)	0.9 (0.6–1.3)	< 0.001
Platelets k per mm^3^, (IQR)	217 (162–279)	192 (150–259)	< 0.001
Alanine aminotransferase (AST) U/L , (IQR)	24 (15–39)	29 (17–44)	0.028
Aspartate aminotransferase (ALT) U/L , (IQR)	34 (23–55)	52 (31–82)	< 0.001
Ferritin µg/L , (IQR)	675.5 (316–1476)	1119 (622–1980)	< 0.001
International normalized ratio (INR), (IQR)	1.1 (1–1.2)	1.1 (1–1.3)	< 0.001
D-dimer mg/ml, (IQR)	1.09 (0.36–2.51)	2.19 (0.35–7.0)	< 0.001
Creatinine µmol/L, (IQR)	1.01 (0.8–1.52)	1.62 (1.03–3.1)	< 0.001
Blood urea nitrogen (BUN) mg/dL, (IQR)	16 (10–28)	29 (13–58)	< 0.001
Glucose mg/dL, (IQR)	132.5 (110–189)	155 (121–232)	0.187
Sodium mmol/L , (IQR)	137 (134–140)	138 (134–142)	< 0.001
Interleukin-6 (IL-6) pg/ml, (IQR)	29.5 (13.7–61.1)	87 (42.3–179.4)	< 0.001
C-Reactive protein (CRP) mg/L , (IQR)	5.5 (1.1–13.6)	13.3 (4.3–23)	< 0.001
Procalcitonin ng/ml, (IQR)	0.2 (0.1–0.5)	0.9 (0.3–3.6)	< 0.001
Troponin ng/ml, (IQR)	0.01 (0.01–0.01)	0.02 (0.01–0.08)	< 0.001

**Table 3:** Multiple logistic regression analysis for in-hospital mortality in the derivation cohort.

Independent Predictors	OR [95% CI] *p* value
**Age (< 60 years-reference)**	
≥ 60 years	2.4 [1.18–5.16] *p* = 0.025
≥ 70 years	3.39 [1.59–7.19] *p* = 0.001
≥ 80 years	5.69 [2.61–12.42] *p* < 0.001
Female sex (*Male sex-reference*)	.95 [.58–1.57] *p* = 0.048
Oxygen saturation < 94% (*≥ 94% -reference*)	2.49 [1.49–4.18] *p* = 0.001
**MAP (> 80 mmHg-reference)**	
≤ 80 mmHg	1.4 [.93–2.12] *p* = 0.109
≤ 70 mmHg	4.34 [2.52–7.48] *p* < 0.001
≤ 60 mmHg	20.53 [4.73–89.0] *p* < 0.001
INR > 1.2 *(≤ 1.2-reference*)	1.24 [.74–2.06] *p* = 0.04
Creatinine > 150 µmol/L (*≤ 150 µmol*/*L-reference*)	1.59 [.83–3.03] *p* = 0.016
BUN > 30 mg/dL (*≤ 30 mg/dL-reference*)	1.53 [.79–2.96] *p* = 0.02
IL-6 > 150 pg/ml (*≤ 150 pg/ml-reference*)	2.06 [1.07–3.96] *p* = 0.03
CRP > 10 mg/L (*≤ 10 mg*/*L-reference*)	1.49 [.85–2.64] *p* = 0.016
Procalcitonin > 0.1 ng/ml (*≤ 0.1 ng/ml-reference*)	4.19 [2.01–8.75] *p* < 0.001

*MAP* mean arterial pressure, *INR* international normalized ratio, *BUN Blood urea nitrogen*, *IL-6* Interleukin-6 *CRP* C-Reactive protein.

The bootstrap analysis revealed that, out of the 10 independent predictors of mortality, age, oxygen saturation, MAP, BUN, CRP, INR and procalcitonin were reproducibly selected in more than 70%. Due to the large number of missing data for procalcitonin (44%), this variable was excluded in order to avoid noise predictors. Allocation of points for the COVID-19 severity score was made based on Beta coefficients and BCa 95%CI, however for the simplicity of the score we allocated points 1 to 3 in subcategorized variables (Age & MAP) (Table 4). The total prediction score ranges between 0 and 10 with a high score indicating high risk of in-hospital mortality.

**Table 4:** Point allocation for predictors of COVID-19 in-hospital mortality.

Scoring factors	Beta coefficient	BCa 95% CI	Score assigned
Age			
≥ 60 years	0.882	0.218–1.67	1
≥ 70 years	1.064	0.434–1.822	2
≥ 80 years	1.500	0.883–2.347	3
Oxygen saturation			
< 94%	0.739	0.285–1.252	1
MAP			
≤ 80 mmHg	0.259	0.428–0.911	1
≤ 70 mmHg	1.43	0.561–2.34	2
≤ 60 mmHg	22.96	21.90–24.31	3
BUN			
> 30 mg/dL	0.495	0.053–1.063	1
CRP			
> 10 mg/L	0.660	0.78–1.069	1
INR			
> 1.2	0.130	0.486–0.743	1
Total score			10

*MAP* mean arterial pressure, *INR* international normalized ratio, *BUN Blood urea nitrogen*, *CRP* C-Reactive protein.

**Figure 1:**
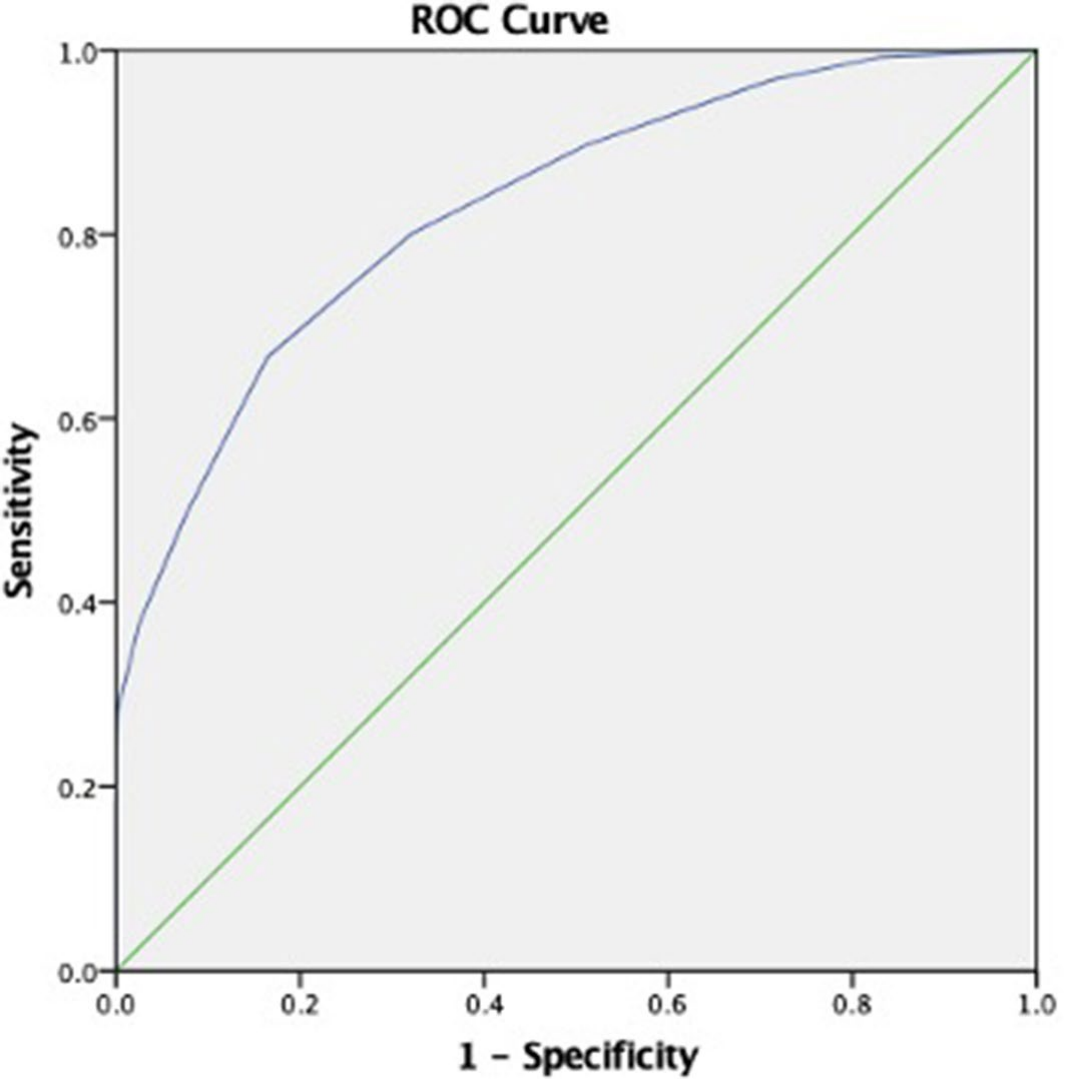
ROC curve analysis in derivation cohort. Area under the curve (AUC) of the COVID-19 in-hospital mortality score in the 2355 patients that constituted the derivation cohort.

A ROC curve analysis was performed in the derivation cohort (Fig. 1), the novel COVID-19 severity score achieved an AUC of 0.824 (95% CI 0.814–0.851) indicating a good discrimination for patients with higher risk of in-hospital mortality. Furthermore, the Hosmer–Lemeshow goodness of fit test of tenfold cross-validation did not reach statistical significance (*P* = 0.244) indicating a good match of predicted risk over observed risk.

**Figure 2:**
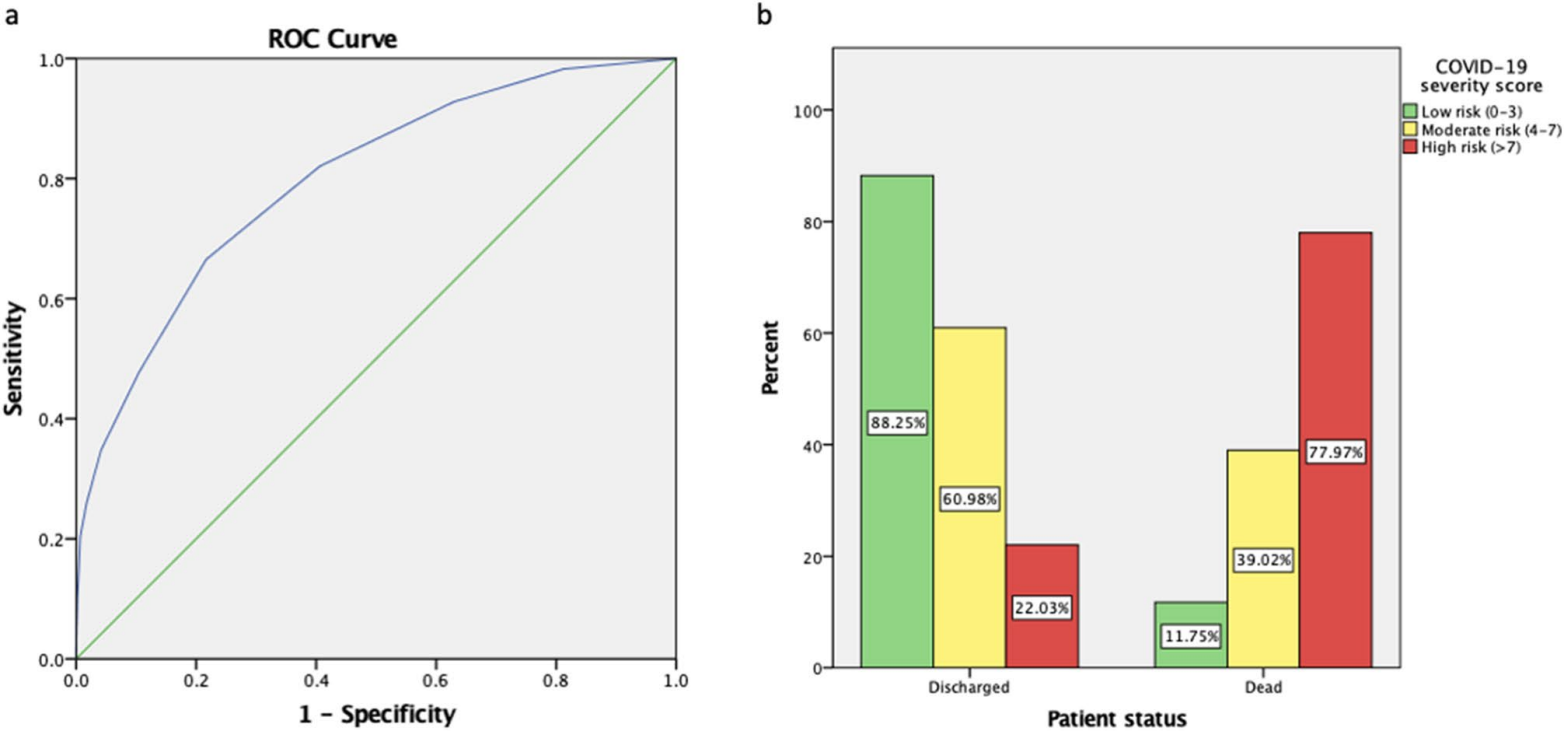
Validation of COVID-19 severity score. (**a**) Area under the curve (AUC) of the COVID-19 in-hospital mortality score in the 2356 patients that constituted the validation cohort. (**b**) Percentage death patients in low, moderate and high-risk categories.

Finally, we applied the score to the 2356 patients in the validation cohort. The ROC curve analysis showed an AUC of 0.798 (95% CI 0.789–0.818) still indicating a useful discrimination for our model (Fig. 2A). Then, we determined that low risk patients (0–3 points) had a 11.8% risk of mortality, moderate risk patients (4–7 points) had a 39% risk of mortality and high-risk patients (> 7 points) had a 78% risk of mortality (Fig. 2B).

## Discussion

We propose a novel scoring system to aid in the prediction of inpatient mortality for patients presenting with SARS-CoV-2 infection to hospital emergency rooms. The score is based on simple pragmatic demographic data, and presenting biomarker values. This score incorporates the unique constellation of various presentations in which COVID-19 can manifest in severe illness. We avoided incorporating mechanical ventilation use into the score as this was tied to a clinical decision, which over time with more knowledge an approach that changed. While IL-6 also seems to predict mortality, we avoided incorporating this biomarker, as it is a non-routine test, and were not available in a large percentage of our patient population. As of yet there are no scoring systems created that are specific to the elements of COVID-19 illness manifestations and that can predict mortality.

The limitations of this study are its retrospective design, its cohort, which is primarily a minority urban population, and the epoch at which the data was required. Since the data and outcomes were recorded during the highest surge of the pandemic this may bias the results towards higher mortality as this was a great strain on treating hospitals at the time. Prior reports also have shown increased mortality in racial and ethnic minority patients^17^. Given the sociodemographic background of our patient population the score may again be biased towards higher mortality risk. While the design of the study may limit its generalizability to other populations, these findings are meaningful in that they are specifically applicable to minority urban centers that are suffering from large surge populations of infected patients, which in the first wave of the pandemic across the United States of America suffered the most. The encountered mortality rate is certainly high, but most likely the result of the high comorbidity burden in our population, the fact that all of these patients had enough symptom severity to warrant admission, and the fact that the study period was early in the pandemic when there was limited understanding regarding the disease. Nonetheless, given the diverse patient population of the Bronx, it is possible that this score can be generalized to other large inner-city populations. Future research is needed to validate this score in other populations, as well as to compare this score to the SOFA and ISTH DICS score. The health network from which this data was captured is comprised of a network of 3 major hospitals in the Bronx in New York City, one of which is a large quaternary care facility accepting transfers for complex and severely ill patients in the region beyond the Bronx into Westchester County. The mortality rates reported here are for hospitalized patients who tended to be older and more severely affected than others infected with the virus. Hence the mortality rate for hospitalized patients is higher than the more commonly reported case-fatality rate that reflects the number of deaths per documented infection. In any case, the rates reported here are broadly comparable to mortality rates for hospitalized patients in other countries at comparable time points in their respective pandemic outbreaks: China—48%^4^, Italy—26%^18^, and New York—21%^19^.

The mortality rates were slightly different between the training set (26.4%) and the testing set (22.4%). This is likely secondary to the temporal difference between the sets. During the first 3 weeks of the pandemic surge, there was still little known about optimal management strategies for severely ill patients. As time went on, mortality rates decreased. In addition, there was more community awareness of the potential impact of the virus and it is possible patients were more likely to seek medical attention sooner and arrived in less severe states. Despite this mortality rate differences, the severity score itself remained valid. There were also variances in racial distribution between the two cohorts. Despite these differences in race, the severity score remained valid in predicting in-hospital mortality.

In other metropolitan areas outside of New York City there have been reports of racial disparity and outcome, we found no difference in mortality rates between races^17,20^. There are a number of possibilities why. The Bronx is uniquely diverse in its racial and ethnic populations however also one of the poorest regions in the United States of America with median income of $38,085 and 27.3% of persons living in poverty^21^. One reason could be that other social determinants of health, including poverty level are more powerful predictors of mortality rather than race alone.

While mortality prediction is neither perfect nor absolute, having a simple score to predict how severe a patient’s illness and hospital course will be, can aid admitting and emergency room physician’s ability to triage severity and predict prognosis during surge periods. This can also be used to guide recommendations for palliative care consultation early in a patient’s hospital course.

## Supplementary information

Supplementary information
