# Supplementary material for: A novel severity score to predict inpatient mortality in COVID-19 patients
Source: Sci Rep. 2020 Oct 7;10:16726. doi: 10.1038/s41598-020-73962-9 (PMC7542454; doi:10.1038/s41598-020-73962-9)
Supplement: Supplementary file 1 — Supplementary information [file 41598_2020_73962_MOESM1_ESM.doc]

**Appendix 1**. References of the studies that have reported laboratory values

| **Laboratory value** | **Reference** |
| --- | --- |
| White Blood Cells (WBC) per mm^3^ | Sise ME, Baggett MV, Shepard JO, Stevens JS, Rhee EP. Case 17-2020: A 68-Year-Old Man with Covid-19 and Acute Kidney Injury. *N Engl J Med*. 2020;382(22):2147-2156. doi:10.1056/NEJMcpc2002418 |
| Lymphocytes per mm^3^ | Sise ME, Baggett MV, Shepard JO, Stevens JS, Rhee EP. Case 17-2020: A 68-Year-Old Man with Covid-19 and Acute Kidney Injury. *N Engl J Med*. 2020;382(22):2147-2156. doi:10.1056/NEJMcpc2002418 |
| Platelets k per mm^3^ | Feldstein LR, Rose EB, Horwitz SM, et al. Multisystem Inflammatory Syndrome in U.S. Children and Adolescents. *N Engl J Med*. 2020;383(4):334-346. doi:10.1056/NEJMoa2021680 |
| Alanine aminotransferase (AST) U/liter | Guan WJ, Ni ZY, Hu Y, et al. Clinical Characteristics of Coronavirus Disease 2019 in China. *N Engl J Med*. 2020;382(18):1708-1720. doi:10.1056/NEJMoa2002032 |
| Aspartate aminotransferase (ALT) U/liter | Feldstein LR, Rose EB, Horwitz SM, et al. Multisystem Inflammatory Syndrome in U.S. Children and Adolescents. *N Engl J Med*. 2020;383(4):334-346. doi:10.1056/NEJMoa2021680 |
| Ferritin µg/liter | Suleyman G, Fadel RA, Malette KM, et al. Clinical Characteristics and Morbidity Associated With Coronavirus Disease 2019 in a Series of Patients in Metropolitan Detroit. *JAMA Netw Open*. 2020;3(6):e2012270. Published 2020 Jun 1. doi:10.1001/jamanetworkopen.2020.12270 |
| International normalized ratio (INR) | Lai X, Wang M, Qin C, et al. Coronavirus Disease 2019 (COVID-2019) Infection Among Health Care Workers and Implications for Prevention Measures in a Tertiary Hospital in Wuhan, China. *JAMA Netw Open*. 2020;3(5):e209666. Published 2020 May 1. doi:10.1001/jamanetworkopen.2020.9666 |
| D-dimer mg/ml | Feldstein LR, Rose EB, Horwitz SM, et al. Multisystem Inflammatory Syndrome in U.S. Children and Adolescents. *N Engl J Med*. 2020;383(4):334-346. doi:10.1056/NEJMoa2021680 |
| Creatinine µmol/liter | Huang C, Wang Y, Li X, et al. Clinical features of patients infected with 2019 novel coronavirus in Wuhan, China [published correction appears in Lancet. 2020 Jan 30;:]. *Lancet*. 2020;395(10223):497-506. doi:10.1016/S0140-6736(20)30183-5 |
| Blood urea nitrogen (BUN) mg/dL | Lai X, Wang M, Qin C, et al. Coronavirus Disease 2019 (COVID-2019) Infection Among Health Care Workers and Implications for Prevention Measures in a Tertiary Hospital in Wuhan, China. *JAMA Netw Open*. 2020;3(5):e209666. Published 2020 May 1. doi:10.1001/jamanetworkopen.2020.9666 |
| Glucose mg/dL | Das S, K R A, Birangal SR, et al. Role of comorbidities like diabetes on severe acute respiratory syndrome coronavirus-2: A review [published online ahead of print, 2020 Aug 3]. *Life Sci*. 2020;258:118202. doi:10.1016/j.lfs.2020.118202 |
| Sodium mmol/liter | Sise ME, Baggett MV, Shepard JO, Stevens JS, Rhee EP. Case 17-2020: A 68-Year-Old Man with Covid-19 and Acute Kidney Injury. *N Engl J Med*. 2020;382(22):2147-2156. doi:10.1056/NEJMcpc2002418 |
| Interleukin-6 (IL-6) pg/ml | Lai X, Wang M, Qin C, et al. Coronavirus Disease 2019 (COVID-2019) Infection Among Health Care Workers and Implications for Prevention Measures in a Tertiary Hospital in Wuhan, China. *JAMA Netw Open*. 2020;3(5):e209666. Published 2020 May 1. doi:10.1001/jamanetworkopen.2020.9666 |
| C-Reactive protein (CRP) mg/liter | Guan WJ, Ni ZY, Hu Y, et al. Clinical Characteristics of Coronavirus Disease 2019 in China. *N Engl J Med*. 2020;382(18):1708-1720. doi:10.1056/NEJMoa2002032 |
| Procalcitonin ng/ml | Huang C, Wang Y, Li X, et al. Clinical features of patients infected with 2019 novel coronavirus in Wuhan, China [published correction appears in Lancet. 2020 Jan 30;:]. *Lancet*. 2020;395(10223):497-506. doi:10.1016/S0140-6736(20)30183-5 |
| Troponin ng/ml | Suleyman G, Fadel RA, Malette KM, et al. Clinical Characteristics and Morbidity Associated With Coronavirus Disease 2019 in a Series of Patients in Metropolitan Detroit. *JAMA Netw Open*. 2020;3(6):e2012270. Published 2020 Jun 1. doi:10.1001/jamanetworkopen.2020.12270 |
